# Rethinking asthma therapy, part 2: transdermal strategies for adjunct asthma and allergy treatments

**DOI:** 10.3389/jpps.2026.15714

**Published:** 2026-03-04

**Authors:** Joseph Correa, Nicole K. Brogden

**Affiliations:** 1 Department of Pharmaceutical Sciences and Experimental Therapeutics, University of Iowa, Iowa City, IA, United States; 2 Department of Dermatology, University of Iowa, Iowa City, IA, United States

**Keywords:** allergy, asthma, drug delivery, microneedle, transdermal

## Abstract

Asthma and allergies affect millions of people globally. Avoiding triggers and allergens is a basic management technique for all asthma subtypes (>80% of asthma patients also suffer from allergies), and pharmacological treatment is the cornerstone for acute exacerbations and ongoing maintenance. Typical treatment options for asthma include inhaled, oral, or injectable dosage forms. However, transdermal drug delivery has great potential to provide an alternative route of administration of necessary asthma and allergy therapies that have traditionally been given in other dosage forms. In Part 1 of this two-part series, we discussed the work done towards incorporating short- and long-acting β2-agonists into transdermal drug delivery systems. Here in part 2, we describe the current literature for transdermal applications of leukotriene antagonists, theophylline, and other adjunct medications that do not fall into one specific drug class. A brief overview of biologics, particularly monoclonal antibodies, and the role in asthma is also included, including some context of transdermal mAb delivery for disease states beyond asthma. Because of the relatedness of asthma and allergies, transdermal applications for allergen immunotherapy is also discussed.

## Introduction

Asthma is a complex pulmonary disorder with many subtypes, impacting more than 300 million patients globally [1]. Disease management is multimodal and includes many drug classes, including β2-agonists, corticosteroids, leukotriene antagonists, long-acting muscarinic antagonists, and other miscellaneous adjunct therapies [1–3]. More recently, monoclonal antibodies (mAbs) have emerged as a treatment option for patients suffering from asthma [4–6]. Disease subtype and severity both impact the selection of individual and combination therapies [1, 3, 7].

Inhalation dosage forms are common for pulmonary disorders, including asthma, but oral drugs are also used as maintenance therapies [3, 8]. However, newer therapies such as mAbs are only available as injectables/infusions [9–11]. Transdermal delivery (absorption of drug through the skin into the systemic circulation) has not been widely considered a viable dosage form for pulmonary disorders. However, transdermal dosage forms can overcome many challenges of inhaled, oral, and injectable products [12, 13]. Physical enhancements such as microneedles (MNs) allow the benefits of transdermal delivery to be further extended to drug molecules that cannot otherwise permeate through the skin [14]. MNs are micrometer-scale projections that create temporary epidermal micropores, allowing a drug to bypass the lipophilic stratum corneum and permeate directly into the dermis [15]. MNs can be used alone or in combination with other physical enhancement approaches such as iontophoresis and ultrasound [13, 16–18].

In Part 1 of this mini-review series, we summarized transdermal studies of short- and long-acting β2-agonists (SABAs and LABAs, respectively), which are typically administered as inhalations. Here we summarize transdermal studies of other asthma treatments, including studies using MNs and other physical enhancements. At the end we briefly cover transdermal allergen-specific immunotherapy, as >80% of asthma patients also suffer from allergies [2].

## Leukotriene antagonists

Montelukast and zafirlukast are commonly prescribed oral leukotriene receptor antagonists [3]. Lowered bioavailability from first pass hepatic metabolism and food effects are challenges with montelukast and zafirlukast, respectively. Commercially available montelukast tablets contain 4–10 mg of drug with ∼64% bioavailability, equating to ∼2.6–6.4 mg delivered systemically [19, 20], while food reduces zafirlukast bioavailability by 40% [21]. We are unaware of transdermal zafirlukast studies, but transdermal montelukast for asthma has been investigated to achieve controlled release for reduced dosing frequency, improved bioavailability, and improved patient compliance [20, 22–24]. A film formulated with sodium alginate and lignosulphonic acid released 100% of loaded montelukast in 3 h; however, cross-linking with barium chloride or calcium chloride and plasticization with glycerine or polyethylene glycol achieved more controlled release [22]. Without plasticizers, cross-linking with barium chloride and calcium chloride resulted in 100% and 50% release, respectively, over 36 h (the cross-linking improved film stability, producing more controlled release). Over the same timeframe with the plasticizers, the films reached 60–80% drug release for barium chloride and 50–70% for calcium chloride.

In another study, montelukast doses in the milligram range were delivered through *ex vivo* porcine skin from a pure-drug MN [20]. A patch containing 3.36 mg of drug delivered an average of 1.5 mg through the skin while just under 1 mg remained in the patch and 0.33 mg remained on the skin. A patch loaded with 5.83 mg of montelukast delivered ∼3.23 mg, which is within the desired range for clinical use [20].

## Theophylline

Theophylline is an oral bronchodilator used in the treatment of asthma and other obstructive pulmonary diseases, also aiding preterm infants needing respiratory support [25–31]. Theophylline doses for asthma management range from 300 to 600 mg/day [32], which is higher than what transdermal dosage forms are typically able to deliver. For this reason, it is possible that transdermal may not be suitable as a first line theophylline dosage form, but chemical and physical permeation enhancements could improve theophylline permeation to the point of being a useful adjunct therapy (particularly for specialized populations).

Transdermal theophylline permeation from suspensions and gels has been well studied *in vitro* and *in vivo*. *In vitro* permeation studies of theophylline prodrug suspensions demonstrated that higher aqueous solubility improves prodrug delivery, increasing flux across mouse skin up to 7 times that of more lipophilic prodrugs [33, 34]. Drug permeation, including theophylline, is often significantly impacted by the composition of transdermal gel formulations [35]. For example, for an ethanol/panasate 800 binary vehicle in an ethylcellulose gel, increasing ethylcellulose concentrations decrease theophylline permeation through mouse skin *in vitro*; however, all tested concentrations of ethylcellulose with the binary vehicle achieved higher permeation than either vehicle independently [36]. In another *in vitro* study, theophylline flux across excised porcine skin was 10 times higher in a water vehicle vs. propylene glycol formulations [37].

### Permeation enhancement

Many permeation enhancers have been studied for transdermal theophylline. Without permeation enhancement, *in vivo* serum theophylline concentrations often fall below the therapeutic range when delivered as a gel [36, 38]. For example, in a 12-cat study, theophylline was loaded into either Lipoderm or pluronic lecithin organogel gel vehicles and applied to cats’ ear skin, achieving serum theophylline levels of 3–4 μg/mL over 24 h on average, which is lower than the 5 μg/mL therapeutic minimum for feline asthma [38].

Ester derivatives improve transdermal theophylline delivery [32–34]. Six-amino hexanoic acid esters increased theophylline permeation across human skin *in vitro* up to 3.9 times vs. negative control; up to 300 μg/cm^2^ of theophylline was delivered over 48 h with 6-amino hexanoic acid esters, reaching flux as high as 9.9 μg/cm^2^ h [39]. ε-aminocaproic acid esters achieved *in vitro* flux across human skin up to 100 μg/cm^2^ h, a 45-fold increase compared to control water vehicle with a flux of 2.2 μg/cm^2^ h [40]. Reactions of dodecyl-6-aminohexanoate with CO_2_ in the air produce a two-chain ammonium carbamate that has further enhanced *in vitro* theophylline permeation through human skin to over 50 times the control, reaching flux up to 176.7 μg/cm^2^ h [41].

Terpenes (naturally occurring compounds often found in essential oils) are another group of permeation enhancers investigated with theophylline [31, 42–45]. Menthol significantly increased theophylline flux across murine skin to 29.15 μg/cm^2^ h vs. 16.78 μg/cm^2^ h without enhancers [31]. In rat skin, higher ethanol concentration slightly improved flux, but terpenes from *Magnolia fargesii* essential oil significantly had a much greater effect, achieving flux of ∼24 μg/cm^2^ h vs. <5 μg/cm^2^ h without terpenes [42]. Addition of oleic acid, menthone, and farnesol to theophylline suspensions significantly increased flux 3.3, 1.9, and 48.1 times over controls, respectively [43, 44]. Lauric acid reduced lag time from 2 to 3 h to <1 h but did not significantly increase theophylline permeation across excised mouse skin *in vitro* after 24 h [36]. However, *in vivo* studies across shaved rat abdomens increased bioavailability from 72% to 99% with lauric acid [36]. The authors attributed these discrepancies to differences between *in vitro* equilibrium-driven permeation and *in vivo* systemic absorption [36]. Other chemical permeation enhancers that have improved theophylline transdermal delivery include sugar-derived and amino acid-derived enhancers and calcium thioglycolate [46–50].

### Physical enhancement

In addition to chemical permeation enhancers, theophylline has been loaded into polymeric MNs for improved *in vitro* transdermal permeation [51]. Compared to a transdermal patch, MN arrays made with 20% Gantrez® AN-139 polymer significantly increased theophylline permeation across porcine skin, delivering 83% of loaded drug vs. 5.5% delivered from the patch over 24 h [51]. The MN arrays had strong mechanical properties with less than 20% needle height reduction and high pore formation in porcine skin with minimal insertion force. In addition to theophylline delivery, MNs could be useful for monitoring theophylline plasma concentrations (a required part of long-term therapy) [52], which presents a non-invasive way to manage chronic therapy needs.

### Specialized populations

Preterm infants and neonates represent a population that could particularly benefit from transdermal theophylline, taking advantage of increased permeability of neonatal skin [26–30]. Evans et al. reported that a hydroxymethyl cellulose gel loaded with 15% theophylline applied to the skin could achieve therapeutic serum theophylline levels in preterm infants [28], though other studies failed to reach therapeutic levels with the same drug loading in glycerin-based gels [29].

## Additional drugs for asthma treatment

Transdermal studies of other miscellaneous asthma therapies have been limited, and these therapeutics largely do not fit into one major group. Such drugs include corticosteroids, glycopyrrolate, and cromolyn [53–59]. Some therapies have also been investigated for topical, intranasal, or ophthalmic administration for local delivery, but those studies are not covered here.

### Corticosteroids

Corticosteroids for asthma include inhaled fluticasone for maintenance therapy and oral prednisone and methylprednisolone for acute episodes [3, 7]. Fluticasone has been loaded into bilosomes for transdermal delivery, which significantly increased flux across rat skin from 1.81 μg/cm^2^ h (aqueous control) to 5.89 μg/cm^2^ h [53]. Prednisone loaded into a propylene glycol gel could not permeate through intact porcine skin, but following laser-microporation, up to 197.18 μg/cm^2^ permeated over 24 h [59]. When loaded into a nanoliposomal sludge with a hydroxypropyl methylcellulose (HPMC) and polyvinyl pyrrolidone (PVP) base, prednisone reached a flux of 26.6 μg/cm^2^ h, which was significantly greater than flux from an aqueous cream, petroleum jelly, or PVP/HPMC gel [57]. Methylprednisolone has also been studied with MN pretreatment; number of MN pretreatments and duration of MN insertion significantly affected amount of methylprednisolone recovered from tissue [55]. It should be noted that prolonged use of topical corticosteroids can lead to many adverse effects including hypopigmentation and skin atrophy, limiting the practical use of topical or transdermal corticosteroids [60, 61].

### Glycopyrrolate

Glycopyrrolate is a long-acting muscarinic antagonist used treat asthma, other pulmonary conditions, and some non-pulmonary indications [54, 56, 62]. Passive glycopyrrolate permeation from water transported 21.49 μg/cm^2^ across porcine skin over 24 h, which increased to 42.23 μg/cm^2^ and 202.25 μg/cm^2^ with MN pretreatment and iontophoresis treatment, respectively. Interestingly, combining iontophoresis with MNs did not further enhance the permeation, delivering 191.04 μg/cm^2^ across porcine skin [56].

### Cromolyn

As a mast cell stabilizer, cromolyn is FDA approved for asthma, nasal allergies, and conjunctivitis; it is also used off-label for food allergies and irritable bowel syndrome [63]. Cromolyn has low absorption from the gastrointestinal tract, prompting development of inhaled, nasal, and ophthalmic dosage forms. Potential transdermal applications were first investigated in 1985, comparing saline solutions of pyranoquinolinedicarboxylic acid derivatives to cromolyn, though transdermal permeation was not specifically investigated [64]. More recently, transdermal cromolyn sodium permeation from ethosomes and liposomes was evaluated. Transdermal flux from ethosomes (18.49 mg/cm^2^ h) was significantly higher than liposomes (1.80 mg/cm^2^ h) and phosphate-buffered saline control (1.18 mg/cm^2^ h) [58].

## Biologics

Despite the many therapies available to treat asthma and allergies, treatment options and outcomes may still not be optimized for patients with severe asthma. Monoclonal antibodies (mAbs) have emerged as specialized, targeted biologic therapies, with six mAbs currently approved for add-on asthma treatments [9, 65] (Table 1). These mAbs are given as injections or infusions [9], which comes with challenges of pain, needle phobia, and the expense and inconvenience of in-clinic administration [15]. Research exploring transdermal mAbs for asthma is currently lacking, likely because passive transdermal delivery is not conducive to the transport of such large molecules. Additionally, mAb treatments for asthma require high doses (ranging from 30 mg to >300 mg), also presenting challenges for passive transdermal delivery.

**TABLE 1:** Comparison of the six approved mAbs for asthma treatment.

mAbs specific considerations	Omalizumab [66–68]	Mepolizumab [69–71]	Reslizumab [72, 73]	Benralizumab [69, 74, 75]	Dupilumab [76, 77]	Tezepelumab [11, 78, 79]
Brand name	Xolair®	Nucala	Cinqair®	Fasenra®	Dupixent®	Tezspire®
Molecular target	IgE	IL-5	IL-5	IL-5 receptor α	IL-4/IL-13	TSLP
Inflammation type treated	Type 2	Type 2	Type 2	Type 2	Type 2	Type 2 and non-type 2
Eosinophil count for greatest efficacy (cells/µL)	≥260	≥150	≥400	≥300	≥150	≥150
Age indication for asthma therapy (years)	≥6	≥6	≥18	≥6	≥6	≥12
Route and dosing	Subcutaneous injection 75–375 mg every 2–4 weeks	Subcutaneous injection 100 mg every 4 weeks (ages ≥12 years) 40 mg every 4 weeks (ages 6–11 years)	Intravenous infusion 3 mg/kg every 4 weeks	Subcutaneous injection 30 mg every 4 weeks for first 3 doses then every 8 weeks (ages ≥12, and ages 6–11 years, ≥35 kg) 10 mg every 4 weeks for 3 doses, then every 8 weeks (ages 6–11 years, <35 kg)	Subcutaneous injection 200 or 300 mg every 2 weeks (after 400 or 600 mg initial dose for ages ≥12 years; loading dose may vary for ages 6–11 years based on weight)	Subcutaneous injection 210 mg every 4 weeks

Technologies such as iontophoresis, ultrasound, and MNs readily permit transdermal protein delivery [10, 12, 80, 81], and there have been recent studies of transdermal biologics for diabetes, hypoglycemia, and osteoporosis [10, 80, 82, 83]. MN delivery of mAbs such as denosumab, bevacizumab, and anti-PBP2a for conditions including cancer, osteoporosis, and corneal neovascularization have also been performed [84–87]. The doses delivered in these studies ranged from 1.1 µg to 10 mg, which leaves much room for improvement given the high mAb doses required for asthma management. A variety of innovations in MN delivery have generally focused on improving the possible range of delivered doses–these innovations include lyophilized reservoirs, powder jet injectors, and micro/nano-particle encapsulation [80, 84, 88, 89]. With further successes and developments, there could be potential for large molecule asthma therapeutics to be developed into non-invasive transdermal systems.

## Microneedles and allergies

Allergens exacerbate symptoms in many patients with allergic asthma [3]. Allergy management aids asthma treatments for these patients, and advances in allergy desensitization, particularly allergen-specific immunotherapy (AIT), have emerged as important strategies [90, 91]. AIT has been used for pollen, molds, and food allergens [91–94] and is often administered subcutaneously (“allergy shots”) or sublingually, improving allergy symptoms and lessening overall medication use [7, 91]. Despite the success of AIT, there are side effects - including redness, injection pain, sneezing, and hives [91, 92, 95]. AIT is not recommended for patients with severe or uncontrolled asthma because serious adverse reactions such as anaphylaxis are more common in those patients [91, 96, 97].

MNs have been explored for providing sustained allergen exposure and targeting skin-specific immune cells (dendritic cells, Langerhans cells) [95, 98]. One focus of MN-mediated immunotherapy has been on peanuts and cow milk [99–104]. Loading allergens into MN arrays does not affect antigenicity but does reduce immune cell infiltration, Th2 cells, and IgE while increasing IgG1, IgG2, TGF-b, and IL-10 levels, all of which contribute to suppressed allergic responses [105–108]. A major benefit of MN-mediated immunotherapy is minimization of side effects that often arise from invasive subcutaneous injections with traditional AIT [100, 103, 104, 106, 109]. While the small size of a MN patch can limit the dose of allergen exposure, doses as low as 0.1–0.5 µg in MN-mediated epicutaneous immunotherapy can produce maximum therapeutic effect in mouse models [90].

## Discussion

Transdermal treatments for asthma and allergies could improve patient compliance and minimize adverse effects by offering unique advantages over traditional inhaled, oral, and injectable routes. Here in Part 2 of this mini-review series, we broadly discussed formulation approaches and physical enhancement techniques to enable transdermal delivery of adjunct asthma therapies and allergy treatments.

Large molecular size and high doses have been long-standing challenges for transdermal delivery, impacting many of the drugs described in this mini-review – specifically, theophylline and mAbs. However, emerging research in physical enhancement methods for protein delivery suggests potential future feasibility for large molecule transdermal delivery. By disrupting the skin barrier, MNs, iontophoresis, and ultrasound show promise in assisting large molecule delivery through skin [10, 80]. Many innovations have also been explored to increase transdermal dosing range, but the field needs continued growth in this area. Commercially available transdermal patches are loaded with up to 40 mg [110, 111], while drug-loaded and drug-coated MNs generally contain up to 10 mg [88, 112]. However, MNs can deliver larger doses up to 33 mg [17, 113], potentially also requiring lower doses to achieve comparable efficacy as other dosage forms [85, 89, 114–116]. Hollow MNs can deliver larger doses than coated or dissolving MNs [117–119], and solid MNs as a pretreatment (followed by application of another formulation over the MN-treated skin) can significantly increase dose delivered [120–122]. Permeation enhancers, applying multiple MN patches, or combination permeation enhancement approaches are additional options to deliver higher doses.

In summary, innovations in formulation, physical and chemical permeation enhancement, and delivery platforms may advance the field towards fully realizing the clinical potential of transdermal systems for asthma and allergy management – including therapeutics that are most often delivered as inhalations or injectables.
